# Using a nomogram based on preoperative serum fibrinogen levels to predict recurrence of papillary thyroid carcinoma

**DOI:** 10.1186/s12885-018-4296-7

**Published:** 2018-04-05

**Authors:** Lei Jianyong, Li Zhihui, Gong Rixiang, Zhu Jingqiang

**Affiliations:** Thyroid and Parathyroid Surgery Group of West China Hospital of Sichuan University, Chengdu, China

**Keywords:** Papillary thyroid carcinoma, Fibrinogen, Lymph node metastasis, Recurrence

## Abstract

**Background:**

Hyperfibrinogenemia is increasingly being recognized as an important risk factor related to cancer stage, development and outcomes. We evaluated whether preoperative serum fibrinogen levels predict recurrence of papillary thyroid carcinoma (PTC).

**Methods:**

We retrospectively collected data for 1023 PTC patients who underwent surgery at our institution from Aug 2014 to Aug 2016. In total, 414 patients (from Aug 2014 to Dec 2015) were used as the training set to build the model, and 609 patients (from Jan 2016 to Aug 2016) were used as the testing set to validate the model.

**Results:**

In the training set, PTC cases with high serum fibrinogen levels were more likely to have multiple PTCs (*P* = 0.001) and to exhibit surrounding tissue or organ invasion (both *P* < 0.01). Moreover, PTC patients with higher serum fibrinogen levels were also more likely to have an advanced tumor stage (T, *P* = 0.001) and distance metastasis (*P* < 0.001), and these patients had a significantly higher rate of postoperative PTC recurrence (*P* = 0.002). All of these findings were validated in the testing set. The results of univariate and multivariate analyses indicated that hyperfibrinogenemia was a risk factor for PTC recurrence. The identified risk factors were incorporated into a nomogram and validated using the testing set (C-index = 0.811, 95% CI: 0.762–0.871).

**Conclusion:**

PTC cases with hyperfibrinogenemia are more likely to have an advanced TNM stage and have a higher rate of PTC recurrence. Our nomogram could be used to objectively and accurately predict PTC recurrence in a clinical setting.

## Background

Papillary thyroid carcinoma (PTC) has rapidly increased in recent years [1, 2], mainly because of the use of ultrasonography (US) and US-guided fine-needle aspiration cytology (FNAC) during preoperative diagnosis [3]. PTC is usually indolent and curable via surgical thyroidectomy followed by TSH suppression or radioiodine treatment. However, lymph node metastases (LNM) develop in approximately 30–80% of PTC patients [4]. LNM increases the risk of locoregional recurrence and may influence cancer-specific survival in some patients with PTC [5]. Because 60–75% of disease recurrences in the neck occur in the lymph nodes, detecting LNM during the initial operation is very important to reduce the reoperation rate and decrease associated risks and complications [6]. However, the sensitivity of preoperative US for diagnosing central compartment lymph node metastasis is low, ranging from 27.3% to 55% [7], mainly because of air in the trachea. Moreover, fine-needle aspiration (FNA) may also be limited to patients diagnosed with central compartment lymph node metastasis because of the risk of recurrent laryngeal nerve injury.

Fibrinogen is a 350-Kda glycoprotein that is synthesized mainly by the liver epithelium [8] and is recognized as one of several acute phase reactant proteins that is produced during systemic inflammation or tissue injury. Fibrinogen that has been converted to insoluble fibrin by activated thrombin can significantly affect blood clotting, the inflammatory response, fibrinolysis, wound healing and neoplasia [9]. Moreover, previous studies have reported that increased fibrinogen activity significantly influences cancer cell growth, progression and metastasis in lung [10], colorectal [11], cervical [12], ovarian [13], esophageal [14], and pancreatic [15] cancer. Furthermore, plasma fibrinogen levels have been associated with tumor size, tumor invasion and lymph node metastasis in a variety of cancers and is recommended as a useful predictor of lymphatic metastasis in gastric cancer [16], non-small cell lung cancer [10], and colorectal cancer [17]. Potential mechanisms of fibrinogen in cancer include angiogenesis stimulation [18], tumor cell proliferation enhancement [19] and immune cell restriction [20]. However, whether serum fibrinogen concentrations represent a significant predictor of tumor stage or recurrence in PTC patients remains unclear and unexplored. Therefore, in the present study, we analyzed the correlations between preoperative serum fibrinogen levels and tumor characteristics and tested the value of using preoperative serum fibrinogen levels as a biomarker for predicting PTC recurrence.

## Methods

### Study design

In the present study, we enrolled 1023 PTC patients, who were divided into the following two independent groups: a training set that comprised 414 consecutive patients and a testing set that comprise 609 consecutive patients with thyroidectomy who were admitted to West China Hospital of Sichuan University (Chengdu, China) between Aug 2014 and Dec 2015 and between Jan 2016 and Aug 2016, respectively. Clinical baseline data were retrospectively collected for each patient from the HIS medical system of our hospital, informed consent was preoperatively obtained from all patients, and this clinical study was approved by the Ethics Committee of West China Hospital. The study was conducted in accordance with the ethical standards of the World Medical Association Declaration of Helsinki. PTC diagnoses, surgical procedures and postoperative follow-up protocols were performed as described in our previous studies [21, 22]: all patients accepted the initial follow-up at 1 month after surgery. The main items investigated were TSH, FT3 and FT4 levels in all cases, and Tg and TgAb in total thyroidectomy cases, which were assessed at the 1-month follow-up, and then every 3 months during the first year followed by every 6 months thereafter. Neck ultrasonography was performed and evaluated in the third month after surgery and then every 6–12 months. A 30 mCi 131I scan was used to detect distant metastasis. We defined PTC recurrence as a structurally incomplete response during the short-term follow-up, as described in the ATA guidelines [2] and other reports [23, 24].

### Inclusion and exclusion criteria

PTC patients who underwent thyroidectomy in our hospital were included in the present study. Patients in this initial group were excluded based on the following criteria: a history of thyroid surgery; other thyroid cancer, such as medullary or follicular thyroid cancer; no data for preoperative plasma fibrinogen level; a concomitant disease suspected of influencing serum fibrinogen concentrations, such as liver fibrosis or other liver disease; acute or chronic renal failure; severe hypertension; coagulation disorder; and anticoagulation therapy within 3 months prior to baseline testing to determine serum fibrinogen levels.

### Serum fibrinogen measurement

A test to determine a patient’s preoperative serum fibrinogen level is a routine and essential test that is performed in all PTC patients who undergo surgery. In this test, 2–3 ml of whole blood is obtained via a peripheral venous puncture 1–3 days before surgery at 07:00 am and evaluated using classical methods (Sysmex XN-9100™). As one of seven coagulation functions, serum fibrinogen concentrations were analyzed as a continuous variable according to the reference value in our hospital, and the normal reference range for plasma fibrinogen concentrations was defined as between 2 and 4 g/L. Hence, hyperfibrinogenemia was defined as a plasma fibrinogen concentration > 4 g/L.

### Univariate and multivariate analysis of PTC recurrence

All factors that were potential risk factors for PTC recurrence were included in the univariate analysis. Preoperative factors, including patient age (≤45 and > 45 or ≤ 55 and > 55); gender (female or male); race (Han, Tibetan or other); smoking (yes/no); alcohol use (yes/no); intraoperative factors, including total thyroidectomy (yes/no) and central compartment lymph node dissection (yes/no); and histological factors, including T stage (T1-T2/T3-T4), N (N1/N0), M (M1/M0), PTC number (single/multiple), bilateral lobe PTC (yes/no), AJCC stage (I-II/III-IV) and postoperative RAI (yes/no). A univariate analysis was used to compare cases with present and absent PTC recurrence. Results with a *P* value equal to or lower than 0.05 were analyzed in a multivariate analysis using Cox’s proportional hazards regression model with a forward stepwise procedure.

### Statistical analysis

All data were entered into an Excel file and then into SPSS. The statistical analysis was performed using SPSS 22.0 for Windows (IBM Corporation, Armonk, USA). Continuous variables are expressed as the mean ± standard deviation, and categorical variables are expressed as percentages (%). A chi-square test and Mann-Whitney U-test were performed to analyze the relationships between preoperative serum fibrinogen levels and PTC recurrence or other clinicopathological variables. Based on the identified risk factors, a nomogram of risk factors associated with PTC recurrence was established in R software studio. The predictive performance of this model was evaluated in the test group using the concordance index (C-index). A *P* value lower than 0.05 in a two-tailed test was considered to indicate a significant difference.

## Results

### Patient characteristics and PTC features

The characteristics of the patients and their tumor features are summarized in Table 1. Alcohol use (*p* = 0.027), BRAF mutation (*p* = 0.030) and T classification (*p* = 0.016) were significantly different between the training set and the testing set, possibly due to the selection process. These differences may also indicate that our predictive model can be universally applied across heterogeneous populations of PTC patients.

**Table 1:** Demographic and Clinicopathological Characteristics of Patients With PTC

	Training set		Testing set		*P* Value
	Patients		Patients		
Factor	No.	%	No.	%	
All patients	414	100	609	100	
Age (years)					0.639
≤45	242	58.5	347	57.0	
>45	172	41.5	262	43.0	
Age (years)					0.079
≤55	357	86.2	500	84.0	
>55	57	13.8	109	16.0	
Gender					0.196
Female	305	73.7	426	82.1	
Male	109	26.3	183	17.9	
Postoperative RAI					0.655
Yes	228	55.1	344	56.5	
No	186	44.9	265	43.5	
Race					0.971
Han	403	97.3	593	97.4	
Tibetan	3	0.7	6	1.0	
Other†	8	1.9	10	1.6	
Smoking					0.603
Never	352	84.5	508	83.4	
Quit	17	4.1	23	3.8	
Ongoing	45	10.9	78	12.8	
Alcohol use					0.027
Never	358	86.0	490	80.5	
Quit	3	0.7	14	2.3	
Ongoing	53	12.8	105	17.2	
BRAF mutation					0.030
Positive	51	12.3	128	21.0	
Negative	77	18.6	88	14.4	
Unknown	286	68.6	393	64.5	
T classification					0.016
T1	160	38.6	197	32.3	
T2	11	2.7	13	2.1	
T3	188	45.4	294	48.3	
T4	55	13.3	105	17.2	
N classification					0.605
N0	196	47.3	293	48.1	
N1a	156	37.7	197	32.3	
N1b	62	15.0	119	19.5	
Distant metastasis					0.361
No	410	99.0	599	98.4	
Yes	4	1.0	10	1.6	
PTC number					0.135
Single	355	85.7	536	88.0	
Multiple	59	14.3	73	12.0	
Bilateral lobe PTC					0.010
Yes	47	11.4	41	6.7	
No	367	88.6	568	93.3	

### Associations between serum fibrinogen levels and clinicopathological features

The serum fibrinogen levels were 2.71 ± 0.57 g/L in the training set and 2.96 ± 0.65 g/L in the testing set. The associations between serum fibrinogen levels and clinicopathological features in PTCs are shown in Table 2. Serum fibrinogen levels were positively correlated with PTC nodule number (*P* = 0.001 in the training set and *P* < 0.001 in the testing set), invasion (including the strap muscles, RLN, esophagus, and trachea; all *P* values were less than 0.01 in both the training and testing group). Moreover, in PTC patients, higher serum fibrinogen levels were associated with advanced tumor stage (T, *P* = 0.001 in both sets) and distant metastasis (*P* < 0.001 in both sets) but not local lymph node metastasis (*P* = 0.186 and *P* = 0.604). Moreover, high preoperative serum fibrinogen levels were significantly associated with a higher incidence of postoperative PTC recurrence (*P* = 0.002 in both sets).

**Table 2:** Relationship between serum fibrinogen levels and clinical characteristics in the training and testing sets of PTC patients

	Training set			Testing set		
	Serum fibrinogen level			Serum fibrinogen level		
Factor	Low	High	*p*	Low	High	*p*
All patients	402	12		575	34	
Age (years)			0.017			0.693
≤45	239	3		328	18	
>45	163	9		247	16	
Age (years)			0.046			0.378
≤55	349	8		474	26	
>55	53	4		101	8	
Gender			0.441			0.394
Female	295	10		400	26	
Male	107	2		175	8	
Postoperative RAI			0.683			0.884
Yes	221	7		324	20	
No	181	5		251	14	
Race			0.215			0.906
Han	392	11		560	33	
Other	10	1		15	1	
BRAF mutation			0.642			0.073
Positive	49	2		125	3	
Negative	74	3		84	4	
Unknown	279	7		366	27	
T classification			**0.001**			**0.001**
T1	160	0		189	8	
T2	8	3		12	1	
T3	181	5		279	15	
T4	53	4		95	10	
PTC nodule number			**0.001**			**<0.001**
Single	345	6		509	22	
Multiple	57	6		66	12	
Capsule invasion			0.068			**0.036**
Presence	194	10		266	22	
Absence	208	2		309	12	
Strap muscles invasion			**<0.001**			**0.013**
Presence	40	6		68	9	
Absence	362	6		507	25	
RLN invasion			**<0.001**			**0.001**
Presence	9	2		44	8	
Absence	393	10		531	26	
Esophagus invasion			**<0.001**			**0.009**
Presence	6	2		18	4	
Absence	396	10		557	30	
Trachea invasion			**<0.001**			**<0.001**
Presence	3	3		11	5	
Absence	399	9		564	29	
N classification			0.186			0.604
N0	193	3		279	14	
N1a	149	7		183	14	
N1b	60	2		113	6	
M classification			**<0.001**			**<0.001**
M0	400	10		569	30	
M1	2	2		6	4	
PTC recurrence			**0.002**			**0.002**
No	373	8		547	28	
Yes	29	4		28	6	
AJCC stage			**0.004**			**0.047**
I	268	4		392	20	
II	62	2		100	3	
III	70	4		77	7	
IV	2	2		6	4	

### The correlation between serum fibrinogen levels and prognosis in PTC patients

**Fig. 1:**
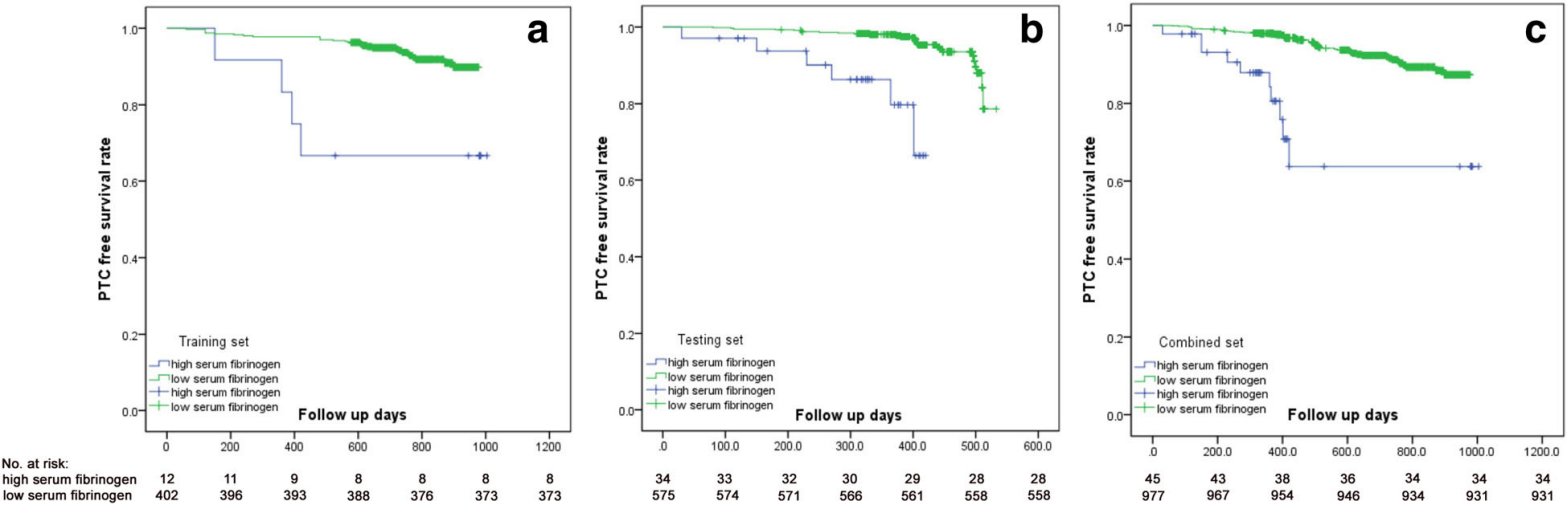
A comparison of postoperative PTC-free survival rates between patients with high and low serum fibrinogen levels. The PTC-free survival rate was much smaller in the high serum fibrinogen level cohort than in the training (**a**, *P* = 0.001), testing (**b**, *P* < 0.001) and combined (**c**, *P* < 0.001) sets

As shown in Table 2, PTC patients with high serum fibrinogen levels had a higher rate of postoperative recurrence. To further investigate the prognostic value of serum fibrinogen in clinical outcomes, we conducted a Kaplan-Meier analysis according to serum fibrinogen levels. A log-rank test was used to compare PTC patients with high and low serum fibrinogen levels in the training, testing and combined sets. Tumor-free survival was significantly lower in patients with high serum fibrinogen levels in the training (as shown in Fig. 1a, *P* = 0.001), testing (Fig. 1b, *P* < 0.001) and combined (Fig. 1c, *P* < 0.001) sets.

### Univariate and multivariate analyses of PTC recurrence

As shown in Table 3, univariate Cox regression analysis revealed that the following clinical factors are significantly associated with PTC recurrence: T classification (T1–2 vs T3–4, *P* = 0.021), N classification (N0 vs N1, *P* = 0.048), M classification (M0 vs M1, *P* < 0.001) and serum fibrinogen level (high vs low, *P* = 0.012) in the training set; and T classification (T1–2 vs T3–4, *P* = 0.008), N classification (No vs N1, *P* = 0.041), M classification (M0 vs M1, *P* < 0.001) and serum fibrinogen level (high vs low, *P* = 0.007) in the testing set. As shown in Table 4, multivariate analysis showed that T/N/M classification and high serum fibrinogen levels were independent prognostic factors of PTC recurrence in the training and testing sets.

**Table 3:** Univariate Cox regression analysis for PTC recurrence in the training and testing patient sets

	Training set		Testing set	
Factor	HR(95% CI)	*p*	HR(95% CI)	*p*
All patients	414		609	
Age (years)		0.622		0.753
≤45	1 (reference)		1 (reference)	
>45	0.982 (0.457 to 1.452)		0.872(0.562 to 1.654)	
Age (years)		0.672		0.261
≤55	1 (reference)		1 (reference)	
>55	1.524 (0.782 to 2.281)		1.622 (0.891 to 2.462)	
Gender		0.321		0.271
Female	1 (reference)		1 (reference)	
Male	1.451 (0.661 to 2.992)		1.652 (0.842 to 2.721)	
Postoperative RAI		0.172		0.283
Yes	1 (reference)		1 (reference)	
No	0.872 (0.521 to 1.726)		0.982 (0.475 to 2.228)	
Race		0.372		0.208
Han	1 (reference)		1 (reference)	
Other	1.211 (0.872 to 1.872)		1.253 (0.772 to 2.011)	
BRAF mutation		0.382		0.229
Positive	1 (reference)		1 (reference)	
Negative	0.871 (0.622 to 1.263)		0.982 (0.821 to 1.461)	
T classification		**0.021**		**0.008**
T1-2	1 (reference)		1 (reference)	
T3-4	3.261 (1.271 to 5.653)		3.876 (1.532 to 6.211)	
PTC nodule number		0.152		0.102
Single	1 (reference)		1 (reference)	
Multiple	1.933 (0.891 to 2.901)		2.011 (0.902 to 3.110)	
N classification		**0.048**		**0.041**
N0	1 (reference)		1 (reference)	
N1	1.502 (1.029 to 2.893)		1.462 (1.081 to 3.011)	
M classification		**<0.001**		**<0.001**
M0	1 (reference)		1 (reference)	
M1	8.662 (3.092 to 16.223)		9.920 (3.825 to 21.273)	
Serum fibrinogen levels		**0.012**		**0.007**
Low	1 (reference)		1 (reference)	
High	3.457 (2.203 to 6.782)		4.228(2.102 to 7.541)

**Table 4:** Multivariate analysis of factors that contributed to PTC recurrence in the training and testing sets

Variables	Odds ratio	95% CI	*P* value
Training set (*n*=414)			
Serum fibrinogen levels (high vs low)	3.152	1.781-5.882	0.002
T classification	4.117	1.340-9.831	<0.001
N classification	1.682	1.132-2.862	0.046
M classification	11.681	5.684-32.391	<0.001
Testing set (*n*=609)			
Serum fibrinogen levels (high vs low)	2.891	1.201-4.874	0.032
T classification	3.872	1.227-10.862	0.001
N classification	1.621	1.201-2.984	0.042
M classification	13.772	4.823-28.910	<0.001

### Nomogram for PTC recurrence

**Fig. 2:**
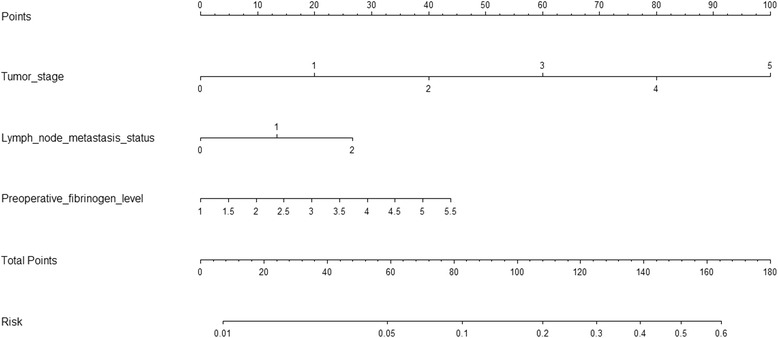
Nomogram for predicting PTC recurrence based on three risk factors:Tumor stages (0, 1, 2, 3, 4 and 5) in Fig. refer to T1a, T1b, T2, T3, T4a and T4b, respectively. The value of each risk factor is respectively loaded on each variable axis (the 2nd-4th lines), and a line is drawn upwards to determine the number of points received for each variable (the 1st line). Then, the sum of these numbers is located on the total points axis (the 5th line), and a line is drawn downwards to the risk axis (the 6th line) to determine the likelihood of PTC recurrence

The risk factors that were found to predict PTC recurrence in the training set were incorporated into the PTC recurrence nomogram. Although AJCC stage is a major risk factor for PTC recurrence, in the nomogram, it was not considered a direct factor. Therefore, we used T classification, N classification and serum fibrinogen levels to build the nomogram, as shown in Fig. 2. Moreover, although metastasis was a risk factor, we did not use it in the nomogram because metastasis is associated with a 100% recurrence rate according to our definition of PTC recurrence. For an individual nodule, the value is loaded on each variable axis (the 2nd-4th lines) and a line is drawn upwards to determine the number of points received for each variable (the 1st line). The sum of these numbers is located on the total points axis (the 5th line), and a line is drawn downwards to the risk axis (the 6th line) to determine the likelihood of PTC recurrence. In the validation cohorts used with the testing set, the C-index in the PTC recurrence nomogram was 0.811 (95% CI, 0.762–0.871). The nomogram further indicated the efficiency of preoperative serum fibrinogen as a predictor of PTC recurrence.

## Discussion

In the present study, we analyzed the correlation between serum fibrinogen levels and tumor stage and found that in PTC, high serum fibrinogen levels (> 4 g/L) are more likely to indicate advanced PTC stage (according to TNM and invasiveness). Moreover, the results of univariate and multivariate analyses indicated that high serum fibrinogen levels are an independent risk factor for PTC recurrence. Finally, using these risk factors, we developed a nomogram to predict the risk of recurrence. To our knowledge, this is the first study to focus on the relationship between serum fibrinogen levels and PTC stage or recurrence with the aim of building a Nomogram. Most importantly, we validated our results in a testing set consisting of a large number of patients. Similar outcomes and conclusions were obtained for the testing and training sets.

Our data show that hyperfibrinogenemia is significantly associated with advanced pathological tumor stage, lymph node metastasis, and adjoining tissue or organ invasion. These findings are consistent with those reported in previous studies examining cancers in other organs [25–27]. Zhang et al. focused on plasma fibrinogen levels in esophageal squamous cell carcinoma and found that patients with hyperfibrinogenemia were more likely to have an advanced pathological T stage, lymph node metastasis and distant metastasis [26]. These results are similar to those reported in studies by Luo et al. that focused on histology in esophageal cancer [25, 28], gastric cancer [29], lung cancer [10] and urothelial carcinoma [30]. In our previous study [31], we reported that hyperfibrinogenemia was associated with a higher rate of lymph node and capsule invasion in medullary thyroid carcinoma. To our knowledge, this is the first study to explore serum fibrinogen levels in PTC. In cancer, hyperfibrinogenemia may also be associated with a higher risk of metastasis, as reported by Zhang et al. [26], who found that esophageal cancer patients with hyperfibrinogenemia exhibited a 2.5-fold increased relative risk of distant metastasis.

In our study, we also show that hyperfibrinogenemia is a risk factor for PTC recurrence. Yamamoto and colleagues [32] compared the efficiency of plasma fibrinogen levels to that of other prognostic markers for predicting gastric cancer recurrence, and their results indicated that plasma fibrinogen level was the most efficient of seven known prognostic markers (the others were carcinoembryonic antigen, carbohydrate antigen 19–9, and C-reactive protein levels, platelet counts, the platelet-to-lymphocyte and neutrophil-to-lymphocyte ratio) for predicting recurrence. Pre-transplant elevated plasma fibrinogen levels constitute a novel prognostic predictor of hepatocellular carcinoma after liver transplantation [33]. Similar correlations have also been observed between hyperfibrinogenemia and recurrence of renal cell carcinoma [34] and other human cancers [35].

Several mechanisms may explain the observed impact of hyperfibrinogenemia. First, fibrinogen may facilitate interactions between cancer and host cells and thereby facilitate metastasis [29]. Second, fibrinogen may help cancer cells evade innate immune cells [20]. Third, a positive feedback loop may exist between fibrinogen and inflammation [36]. Fourth, fibrinogen may enhance tumor progression by inducing tumor cell proliferation, migration and angiogenesis [18, 19]. Finally, fibrinogen surrounding tumor cells may serve as a scaffold that binds members of growth factor families, which may contribute to tumor proliferation and stimulate angiogenesis [37].

We developed a nomogram based on the identified risk factors because a nomogram allows data to be more easily visualized and used in a clinical setting. This nomogram may also be useful for designing follow-up protocols or postoperative adjuvant therapy regiments, such as TSH suppressive therapy or 131I radioactive therapy. Moreover, this model may facilitate communication between surgeons and their patients or the families of patients regarding prognostic analyses and postoperative sequential therapy [38]. To use this nomogram, a PTC patient’s value is located on the corresponding variable axis, and a vertical line is drawn upwards to obtain the number of total points. All of the points for the variable are then added, and then the sum of these numbers is allocated in the Risk axis. A prediction regarding the possibility of PTC recurrence can thereby be calculated in every PTC case.

However, this study has several limitations. First, this is a retrospective research study that focused on data from a single center, which may had resulted in selection bias. However, the large size of the PTC patient cohort may have reduced this bias. Moreover, we select 609 cases to use as a testing cohort to effectively validate our results. Second, our results were only validated using data from our center, and this may limit its usefulness in other centers. However, we are currently performing a study using data across multiple centers in the hope of obtaining more objective results. Last, we cannot provide information regarding recurrence site due to the retrospective study design as most of this information was not recorded.

## Conclusion

In conclusion, we found that hyperfibrinogenemia is highly correlated with advanced TNM stage and a higher recurrence rate in PTC patients. Our nomogram, which was based on the risk factors identified in this study, objectively and accurately predicted PTC recurrence.

## Abbreviations

ATA: American Thyroid Association
LNM: Lymph node metastasis
PTC: Papillary thyroid carcinoma
US: Ultrasonography
